# New insights on the pathogenesis of paraganglioma and pheochromocytoma

**DOI:** 10.12688/f1000research.14568.1

**Published:** 2018-09-20

**Authors:** Vitaly Kantorovich, Karel Pacak

**Affiliations:** 1 Section of Endocrine, MedStar Washington Hospital Center, Washington, DC, USA; 2 Section on Medical Neuroendocrinology, Eunice Kennedy Shriver National Institute of Child Health and Human Development, National Institutes of Health, Bethesda, MD, USA

**Keywords:** Pheochromocytoma, paraganglioma, PPGL

## Abstract

Pheochromocytomas (PCCs) and paragangliomas (PGLs) are rare chromaffin cell tumors (PPGLs) that at times raise significant challenges in clinical recognition, diagnosis, and therapy and when undiagnosed could associate with severe morbidity. Recent discoveries in PPGL genetics propelled our understanding in the pathophysiology of tumorigenesis and allowed the application of functional classification of pathogenetically distinct groups of PPGLs. This also resulted in a qualitative change in our approach to clinical assessment, diagnosis, and therapy of different subgroups of PPGLs. Establishment of the fact that mutations in multiple components of the PHD–VHL–HIF-2α pathway associate with pseudohypoxia-driven tumorigenesis allowed us not only to better understand the effect of this phenomenon but also to more deeply appreciate the value of functional abnormalities in the physiologic tissue oxygen-sensing mechanism. Mutations in the tricarboxylic acid cycle–related genes opened an additional window into understanding the physiology of one of the basic cellular metabolic pathways and consequences of its disruption. Mutations in the kinase signaling–related genes allow the PPGL field to join a massive innovative process in therapeutic advances in current oncology. New pathophysiologically distinct groups of mutations will widen and deepen our understanding of additional pathways in PPGL tumorigenesis and hopefully introduce additional diagnostic and therapeutic approaches. All of these developments are tremendously important in our understanding of both the normal physiology and pathophysiology of PPGLs and are strong tools and stimuli in the development of modern approaches to all components of medical management.

## Introduction

Pheochromocytomas (PCCs) and paragangliomas (PGLs) are chromaffin cell tumors derived from adrenal (PCC) or extra-adrenal sympathetic or parasympathetic paraganglia (PGL) ^1^, respectively. Though once thought of as being mostly benign and biochemically active, these tumors could show a wide spectrum of cellular and biochemical de-differentiation, including aggressive metastatic course and biochemical silence. Despite being anatomically distinct, PCCs and PGLs display a common pathologic basis and frequently are referred to as pheochromocytoma/paraganglioma tumors (PPGLs), the term we will use throughout this article. The incidence and prevalence of PPGLs are based on older literature, which in part paralleled our understanding of the field and could be misleading to some degree. Overall, it is stated that the annual incidence of PCC is between two and eight cases per million, whereas in patients with hypertension, the prevalence of PPGL varies between 0.2 and 0.6%. It is important to remember that in patients with incidentally radiologically discovered adrenal masses, 5% will have PCCs (reviewed in 1, 2).

Recent years have tremendously changed our understanding of each and every aspect of PPGL biology—genetics, clinical and biochemical behavior, pathogenesis, diagnosis, and treatment—which to a major degree also propelled our understanding of PPGL-related and general pathophysiology of tumorigenesis. The PPGL field has also undergone a significant transformation in recent years. In older times, pheochromocytoma was seen as a mostly sporadic and benign disease, usually seen by endocrinology and surgery. Hereditary cases were relatively rare and included MEN2, neurofibromatosis type 1, and von Hippel–Lindau syndromes. Sympathetic paragangliomas were usually managed by the anatomically related (chest, abdomen, and pelvis) surgical specialties with significantly more frequent involvement of medical oncology and endocrinology on an “as needed basis”. Parasympathetic silent head-and-neck paragangliomas were seldom seen as part of the PPGL syndrome and were managed by ear, nose, and throat (ENT) and oncology. With increasing understanding of the high heritability of PPGL and similarity in pathology of seemingly discrete conditions, the field has transformed into all-inclusive and comprehensive medicine approach-based multispecialty management. We now know that PPGLs represent the highest hereditary-driven endocrine condition—up to 40% of cases are related to mutations in 15 well-established driver genes and a growing number of disease-modifying genes and these numbers are expected to grow ^3– 5^.

**Figure 1.**
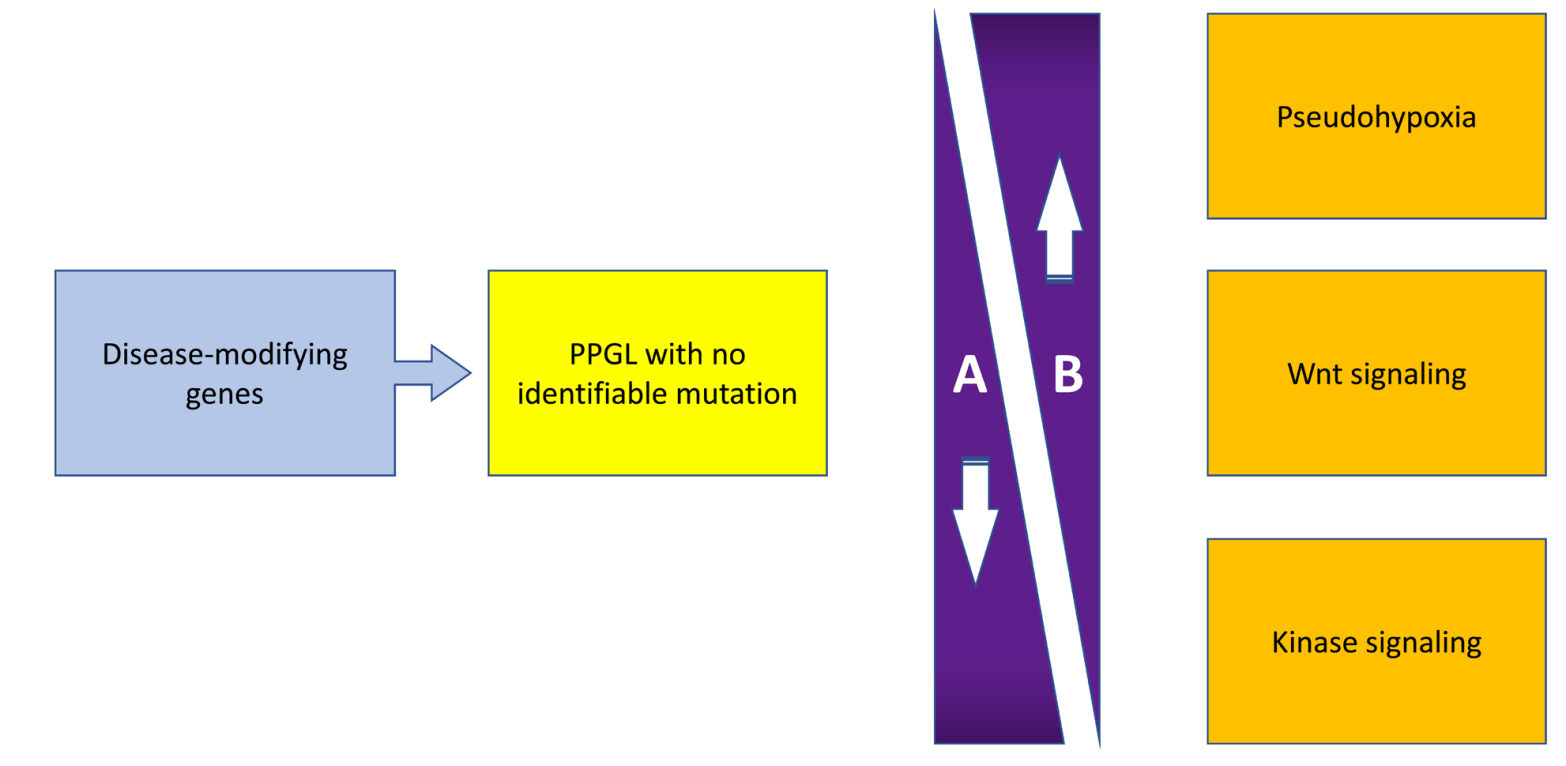
Differential characteristics of hereditary pheochromocytoma/paraganglioma tumors (PPGLs). **A**, degree of PPGL cell differentiation; **B**, risk of metastatic/malignant potential and norepinephric versus epinephric secretory profile.

As our understanding of PPGLs deepens, it seems that current anatomic (World Health Organization, 2017) or standard staging (American Joint Commission Cancer staging, 2017) classifications fell short of providing sufficient insight into pathogenesis, clinical presentation, and (perhaps more importantly) prognostic value with respect to malignant potential, response to therapy, and possible recurrences. Functional, pathogenesis-based classification does allow a better understanding of actual tumorigenesis, expected biochemical profiles, tumor location, and malignant potential. The more recently formulated group of disease-modifying genes is expected to expand and possibly explain tumor formation in a growing number of apparently mutation-free cases ( Figure 1 and Table 1).

**Table 1.** Characterization of driver genes.

Pseudohypoxia group genes					
	Inheritance	H&N PGL	SPGL	PCC	Other manifestation
*SDHA*	AD	Very low	Very low	Very low	Gastrointestinal stromal tumor, pituitary tumors, renal cell cancer, and pulmonary chondroma
*SDHB*	AD	Intermediate	Intermediate	Low	Same
*SDHC*	AD	Low	Low	Low	Same
*SDHD*	AD paternal	High	low	Low	Same
*SDHAF2*	AD	High	Very low	Very low	Same
*FH*	AD	Unknown	Unknown	Unknown	Leiomyomatosis and renal cell cancer
*VHL*	AD	Very low	Low	High	Hemangioblastoma, renal cell cancer, and pancreatic neuroendocrine tumors
*EPAS1*	Unknown	Very low	High	High	Polycythemia, somatostatinoma, and retinal abnormalities
Wnt signaling pathway genes					
*CSDE1*—Detected only as somatic mutations					
*MAML3*—Detected only as somatic mutations					
Pseudohypoxia group genes					
*RET*	AD	Very low	Very low	High	Multiple endocrine neoplasia syndromes
*NF1*	AD	Very low	Very low	Low	Neurofibromas, dysmorphic features, skeletal abnormalities, and so on
*MAX*	AD	Unknown	Unknown	Unknown	Renal oncocytoma
*TMEM127*	AD	Very low	Low	Intermediate	
*HRAS*—Detected only as somatic mutations					

AD, autosomal dominant; H&N PGL, head-and-neck paraganglioma; PCC, pheochromocytoma; SPGL, sympathetic paraganglioma.

This functional classification of currently established susceptibility genes consists of four major groups: the pseudohypoxia group, the kinase signaling group, the Wnt signaling group, and the disease-modifying gene group.

## Pseudohypoxia group

This group can be further subdivided to the following subgroups:

### *VHL/EGLN1/EPAS1*-related

Historically, this group was the first window into our understanding of the pathophysiology of the development of PPGLs. It represents 15 to 20% of PPGLs, which are hereditary in 25% of cases, and includes tumors associated with mutations in von Hippel–Lindau tumor suppressor gene ( *VHL*), Elegans homologs ( *EGLN1–3*), and endothelial PAS domain protein 1 ( *EPAS1*) gene. *VHL*, expressing both germline and somatic mutation patterns, shows mutation in up to 100% of patients with *VHL*-related PPGL. The product of *VHL* is a von Hippel–Lindau tumor suppressor protein (pVHL), which serves as a component of an E3 ubiquitin ligase complex. The mechanism of tumorigenesis in this condition is related to the generation of the pseudoxypoxic metabolic state related to the stabilization of the hypoxia-inducible factor (HIF) (reviewed in 6). HIF is a heterodimer protein which consists of two subunits: HIFα and HIFβ. The first is regulated by oxygen concentration and consists of three isoforms. Under normal conditions, HIF-1α and HIF-2α are rapidly hydroxylated by propyl hydroxylase domain proteins (PDH1–3), which allows them to become recognized by the VHL and delivered to proteasome for degradation. Lack of rapid degradation of the HIFα occurs in states of true hypoxia and pseudohypoxia. Whereas the first will be self-limited and seldom provide enough time for efficient tumorigenesis, pseudohypoxia can definitely extend to the significant length of time; in the case of *VHL* mutation, it will associate with a lack of recognition and degradation of the HIFα and β heterodimer (in the case of PPGL, it is mostly HIF-2α), which will bind to the core DNA RCTCG sequence at the hypoxia-responsive elements (HREs) to activate transcription of multiple genes that could associate with tumor formation, including glucose transporter 1 (GLUT1), vascular endothelial growth factor (VEGF), and many others.

The importance of the PHD–VHL–HIF-2α pathway in the pathogenesis of PPGL was also shown through the association of mutations in every component of this pathway (reviewed in 7). Mutations in *EGLN* 1 and 2, which encode PDH 2 and 1, respectively, were also shown to associate with the development of PPGLs.

Germline, mosaic, or constitutional gain-of-function mutations in *EPAS1* also associate with an increase in the transcriptional activity of HIF-2α target genes ^8, 9^, although the actual transcriptional signature seems to differ somewhat from the one associated with VHL and is associated with a distinct clinical constellation, including PPGL, polycythemia, and somatostatinomas, and is referred to as Zhuang–Pacak syndrome.

### Tricarboxylic acid cycle–related

These are germline truncating mutations in succinate dehydrogenase ( *SDH*) subunits *A*, *B*, *C*, and *D*; *SDHAF2* assembly factor for *SDH*; and fumarate hydroxylase ( *FH*). These tumors represent about 10 to 15% of all PPGLs, are close to 100% hereditary, and functionally represent a tumor suppressor gene mutation-related tumorigenesis. SDH is a dual-functional mitochondrial enzyme, which converts succinate to fumarate in tricarboxylic acid (TCA), and participates as a complex II in the respiratory electron transfer within the mitochondrial membrane. FH subsequently converts fumarate to malate within the same TCA cycle (reviewed in 2, 10, 11). There are recently described constitutional mutations of the malate dehydrogenase ( *MH2*)—the enzyme that converts malate to oxaloacetate further down the TCA—that associate with PGL in a single family ^12^. The exact mechanism of tumorigenesis is thought to be related to oncogenic properties of accumulated TCA metabolites—succinate, fumarate, and malate—by inhibiting enzymes involved in cell signaling and chromatin maintenance ^13^. Succinate and fumarate can inhibit PDH, thus activating the HIF-related tumorigenesis pathway. In addition, both can drive epigenomic tumorigenesis through dose-dependent DNA hypermethylation by inhibition of α-ketoglutarate–dependent oxidative demethylation ^10^.

## Kinase signaling group

This group comprises the majority of PPGLs (50 to 60%) and is driven by either germline or somatic mutations in up to 20% of cases. It includes oncogenes—rearranged during transfection ( *RET*) proto-oncogene and H-ras GTPase proto-oncogene ( *HRAS*)—and several tumor suppressors: neurofibromin 1 ( *NF1*), transmembrane protein 127 ( *TMEM127*), and MYC-associated factor X ( *MAX*). These mutations are associated with the activation of kinase signaling pathways: RAS-RAF-MEK and PI3K-AKT-mTOR for *RET*, *HRAS*, *NF1*, and *TMEM127* and MYC-MAX for *MAX*.

Activation of *RET* gene is essential for development of both sympathetic and parasympathetic components of the autonomous nervous system through growth and differentiation of the neural crest precursors. Tumorigenesis occurs through activation of the tyrosine kinase, which signals through the above pathways to promote cell growth and survival. The *NF1* gene is predominantly expressed in neurons, Schwan cells, oligodendrocytes, and leukocytes. Its main function as a tumor suppressor is ras GTP-ase which de-activates RAS, thus preventing activation of the oncogenic RAS-RAF-MEK signaling cascade. *TMEM127* is a negative regulator of mTOR, which is a PI3K kinase, shown to be activated in many human cancers and associated with increased cell proliferation, angiogenesis, and survival. MAX is a transcription factor associated with proto-oncogene *MYC*.

Rat sarcoma viral oncogene ( *ras*) genes are frequently found in human cancers and function as activators of cell growth and survival. Of multiple group members, only Harvey ( *H-ras*) and Kirsten ( *K-ras*) were shown to associate with PPGL formation.

A small proportion of PPGLs show additional kinase signaling pathway–associated mutations in *FGFR1*, *KIF1B*, and *MET* but these still need to be validated. *KIF1B* is a tumor suppressor gene necessary for the neuronal apoptosis.

## Wnt signaling group

Tumors in this group occur in 5 to 10% of PPGLs and in most cases are related to somatic mutations in tumor suppressor cold shock domain-containing E1 ( *CSDE1*) and oncogene mastermind-like transcriptional coactivator 3 ( *MAML3*).

## Disease-modifying gene group

Somatic mutations in five genes— *ATRX*, *KMT2D*, *SETD2*, *TERT*, and *TP53*—have a synergistic effect on PPGL tumorigenesis and raise the possibility of association with an excessively aggressive course of the disease.

Recent advances in our understanding of the pathogenesis of PPGL had significantly changed our approach to the condition. The change in classification moved different forms of PPGLs into pathogenesis-driven groups, which allowed a better understanding of major disease-related features: clinical presentation, biochemical approach, and predisposition to malignant course and possible differences in therapy and follow-up. It had also brought back some old concepts—like Warburg effect (tumorigenesis related to accelerated glycolysis)—that fit well into the model of pseudohypoxia-driven disease. Understanding differential pathogenetic pathways in different forms of PPGL allowed a better design of biochemical tests with a recent use of metabolomics through high-end nuclear magnetic resonance spectroscopy as a differential tool on a biochemical level ^14, 15^. Pathogenesis-based knowledge had also allowed us to design new therapeutic approaches; the best example would be the use of specific HIF-2α antagonists (PT2399) and prolyl hydroxylase activators (R59949 and KRH102053) that promote HIF hydroxylation, thus restoring VHL-driven recognition and rapid degradation. These could switch the pseudoxypoxic signal and potentially disrupt the tumorigenesis process at the actual pathogenetic point ^15^. The possibility of multiple additional clinical presentations and novel mutations will further boost our ability to more deeply understand and affect the pathogenesis of this fascinating condition. One example would be a recent report of PPGL associated with cardiac dysplasia ^16^. There are still plenty of questions waiting to be answered in relation to the particular mechanisms of tumorigenesis and possible interventions early in the course of the disease to prevent possible adverse outcomes. This will definitely improve with new knowledge and experience.

## References

[1] Lenders JW Duh QY Eisenhofer G: Pheochromocytoma and paraganglioma: an endocrine society clinical practice guideline. *J Clin Endocrinol Metab.* 2014;99(6):1915–42. 10.1210/jc.2014-1498 24893135

[2] Crona J Taïeb D Pacak K: New Perspectives on Pheochromocytoma and Paraganglioma: Toward a Molecular Classification. *Endocr Rev.* 2017;38(6):489–515. 10.1210/er.2017-00062 28938417 PMC5716829

[3] Dahia PL: Pheochromocytoma and paraganglioma pathogenesis: learning from genetic heterogeneity. *Nat Rev Cancer.* 2014;14(2):108–19. 10.1038/nrc3648 24442145

[4] Fishbein L Leshchiner I Walter V: Comprehensive Molecular Characterization of Pheochromocytoma and Paraganglioma. *Cancer Cell.* 2017;31(2):181–93. 10.1016/j.ccell.2017.01.001 28162975 PMC5643159

[5] Favier J Amar L Gimenez-Roqueplo AP: Paraganglioma and phaeochromocytoma: From genetics to personalized medicine. *Nat Rev Endocrinol.* 2015;11(2):101–11. 10.1038/nrendo.2014.188 25385035

[6] Jochmanová I Yang C Zhuang Z: Hypoxia-inducible factor signaling in pheochromocytoma: turning the rudder in the right direction. *J Natl Cancer Inst.* 2013;105(17):1270–83. 10.1093/jnci/djt201 23940289 PMC3888279

[7] Pillai S Gopalan V Smith RA: Updates on the genetics and the clinical impacts on phaeochromocytoma and paraganglioma in the new era. *Crit Rev Oncol Hematol.* 2016;100:190–208. 10.1016/j.critrevonc.2016.01.022 26839173

[8] Zhuang Z Yang C Lorenzo F: Somatic *HIF2A* gain-of-function mutations in paraganglioma with polycythemia. *N Engl J Med.* 2012;367(10):922–30. 10.1056/NEJMoa1205119 22931260 PMC3432945

[9] Pacak K Jochmanova I Prodanov T: New syndrome of paraganglioma and somatostatinoma associated with polycythemia. *J Clin Oncol.* 2013;31(13):1690–8. 10.1200/JCO.2012.47.1912 23509317 PMC3807138

[10] Castro-Vega LJ Buffet A De Cubas AA: Germline mutations in *FH* confer predisposition to malignant pheochromocytomas and paragangliomas. *Hum Mol Genet.* 2014;23(9):2440–6. 10.1093/hmg/ddt639 24334767

[11] Letouzé E Martinelli C Loriot C: SDH mutations establish a hypermethylator phenotype in paraganglioma. *Cancer Cell.* 2013;23(6):739–52. 10.1016/j.ccr.2013.04.018 23707781

[12] Cascón A Comino-Méndez I Currás-Freixes M: Whole-exome sequencing identifies *MDH2* as a new familial paraganglioma gene. *J Natl Cancer Inst.* 2015;107(5): pii: djv053. 10.1093/jnci/djv053 25766404

[13] Selak MA Armour SM MacKenzie ED: Succinate links TCA cycle dysfunction to oncogenesis by inhibiting HIF-alpha prolyl hydroxylase. *Cancer Cell.* 2005;7(1):77–85. 10.1016/j.ccr.2004.11.022 15652751

[14] Rao JU Engelke UF Sweep FC: Genotype-specific differences in the tumor metabolite profile of pheochromocytoma and paraganglioma using untargeted and targeted metabolomics. *J Clin Endocrinol Metab.* 2015;100(2):E214–22. 10.1210/jc.2014-2138 25459911 PMC5393507

[15] Tella SH Taïeb D Pacak K: HIF-2alpha: Achilles' heel of pseudohypoxic subtype paraganglioma and other related conditions. *Eur J Cancer.* 2017;86:1–4. 10.1016/j.ejca.2017.08.023 28946040 PMC6287501

[16] Vaidya A Flores SK Cheng ZM: EPAS1 Mutations and Paragangliomas in Cyanotic Congenital Heart Disease. *N Engl J Med.* 2018;378(13):1259–61. 10.1056/NEJMc1716652 29601261 PMC5972530

